# Subcellular proteomics combined with bioenergetic phenotyping reveals protein biomarkers of respiratory insufficiency in the setting of proofreading-deficient mitochondrial polymerase

**DOI:** 10.1038/s41598-020-60536-y

**Published:** 2020-02-27

**Authors:** Kelsey L. McLaughlin, Kimberly A. Kew, Joseph M. McClung, Kelsey H. Fisher-Wellman

**Affiliations:** 1 Department of Physiology, Brody School of Medicine, East Carolina University, Greenville, NC 27834 USA; 2 Department of Biochemistry and Molecular Biology, Brody School of Medicine, East Carolina University, Greenville, NC 27834 USA; 3 Department of Cardiovascular Sciences, Brody School of Medicine, East Carolina University, Greenville, NC 27834 USA; 4 East Carolina Diabetes and Obesity Institute, East Carolina University, Greenville, NC 27834 USA

**Keywords:** Proteomics, Mitochondria, Energy metabolism, Ageing

## Abstract

The mitochondrial mutator mouse is a well-established model of premature aging. In addition to accelerated aging, these mice develop hypertrophic cardiomyopathy at ~13 months of age, presumably due to overt mitochondrial dysfunction. Despite evidence of bioenergetic disruption within heart mitochondria, there is little information about the underlying changes to the mitochondrial proteome that either directly underly or predict respiratory insufficiency in mutator mice. Herein, nLC-MS/MS was used to interrogate the mitochondria-enriched proteome of heart and skeletal muscle of aged mutator mice. The mitochondrial proteome from heart tissue was then correlated with respiratory conductance data to identify protein biomarkers of respiratory insufficiency. The majority of downregulated proteins in mutator mitochondria were subunits of respiratory complexes I and IV, including both nuclear and mitochondrial-encoded proteins. Interestingly, the mitochondrial-encoded complex V subunits, were unchanged or upregulated in mutator mitochondria, suggesting a robustness to mtDNA mutation. Finally, the proteins most strongly correlated with respiratory conductance were PPM1K, NDUFB11, and C15orf61. These results suggest that mitochondrial mutator mice undergo a specific loss of mitochondrial complexes I and IV that limit their respiratory function independent of an upregulation of complex V. Additionally, the role of PPM1K in responding to mitochondrial stress warrants further exploration.

## Introduction

The mitochondrial polymerase gamma (Polg) is responsible for both replicating and maintaining the fidelity of the mitochondrial DNA (mtDNA)^1^. Disruption of the proofreading capacity of this enzyme complex has been shown to lead to an accumulation of single point mutations and deletions throughout the mitochondrial genome^2–5^. The mtDNA mutator mouse is a genetic knock-in model in which mice express a proofreading-deficient Polg harboring a D257A mutation, thus increasing mutational burden of the mtDNA^2,4^. Mice homozygous (D257A^+/+^) for this transgene display a progeroid phenotype characterized by kyphosis, hair loss, hearing loss, pronounced cardiomyopathy, and reduced lifespan compared to wild-type littermates^2,4,6^.

At the cellular level, increased mtDNA mutational load and/or overt respiratory dysfunction has been associated with increased levels of reactive oxygen species, possibly due to a high amount of electron leak from the respiratory system^7–9^. Our lab and others have demonstrated profound bioenergetic limitations in mitochondria isolated from adult D257A^+/+^ tissues^2,4,10^. Recently we found that respiratory conductance, a measure of bioenergetic efficiency, across a span of physiological ATP free energy demand states, was globally suppressed in D257A^+/+^ heart mitochondria^10^. Although not discussed in the original publication^10^, we observed considerable variability in the absolute conductance across all samples, despite clear group differences in respiratory conductance between WT and D257A+/+ mice. Given that all mice were aged to ~13 months, we reasoned that mice with the most severe decreases in respiratory flux may also present with the greatest decreases in respiratory complex expression. That said, it is currently unknown which, if any, of the hundreds of subunits that comprise the respiratory system would be most predictive of overall biochemical conductance. When determining respiratory complex expression via western blot, a clear decrease in the abundance of complex I (CI) and complex IV (CIV) in D257A^+/+^ mitochondria was observed^10^. Although reduced CI and CIV expression has been previously reported in heart^5^, liver^5^, skeletal muscle^11^, and brain^11,12^ of D257A^+/+^ mice, only one of these studies has expanded upon these findings using mitochondria-enriched mass-spectrometry-based proteomics^12^. Furthermore, no studies have linked changes in mitochondrial function observed in D257A^+/+^ mice to changes across the mitochondrial proteome, including an assessment of both respiratory and non-respiratory proteins.

The objective of the present study was twofold; 1) to associate biochemical measurements of mitochondrial function to respiratory subunit expression, and 2) to investigate potential adaptations across the mitochondrial proteome (both respiratory and non-respiratory) that contribute to or predict respiratory insufficiency in D257A^+/+^ mice. Proteomics experiments were conducted using nLC-MS/MS in mitochondria isolated from heart and skeletal muscle. Heart mitochondria-enriched proteomic data were then correlated with substrate-independent respiratory conductance. Correlations between respiratory conductance and protein abundance were expected to indicate potential biomarkers of respiratory insufficiency related to compromised mtDNA fidelity.

## Results

### Proofreading-deficient mitochondrial DNA polymerase lowers CI and CIV expression in heart and skeletal muscle

To investigate the proteomic impact of chronic bioenergetic insufficiency caused by the accumulation of mtDNA mutations, label-free quantitative nLC-MS/MS was performed on cardiac mitochondria from D257A^+/+^ mice and WT littermates. This approach yielded 1,579 proteins identified and quantified across all samples (Supplemental Fig. 1A). Importantly, the top 10 most abundant proteins corresponded to known mitochondrial proteins (ATP5F1A, ATP5F1B, ACO2, IDH2, MDH2, HADHA, HADHB, ACAA2, UQCRC2, SDHA), confirming enrichment of the mitochondrial proteome using differential centrifugation. In support of this, over half of the total protein intensity, per sample, was attributable to mitochondrial proteins (assigned according to the MitoCarta 2.0 database; Supplemental Fig. 1B). Compared to WT controls, 167 proteins were differentially expressed (adjusted p value <0.1) in D257A^+/+^ cardiac mitochondria, 107 corresponding to the mitochondrial proteome (Supplemental Fig. 1C, Supplementary Table 1).

**Figure 1:**
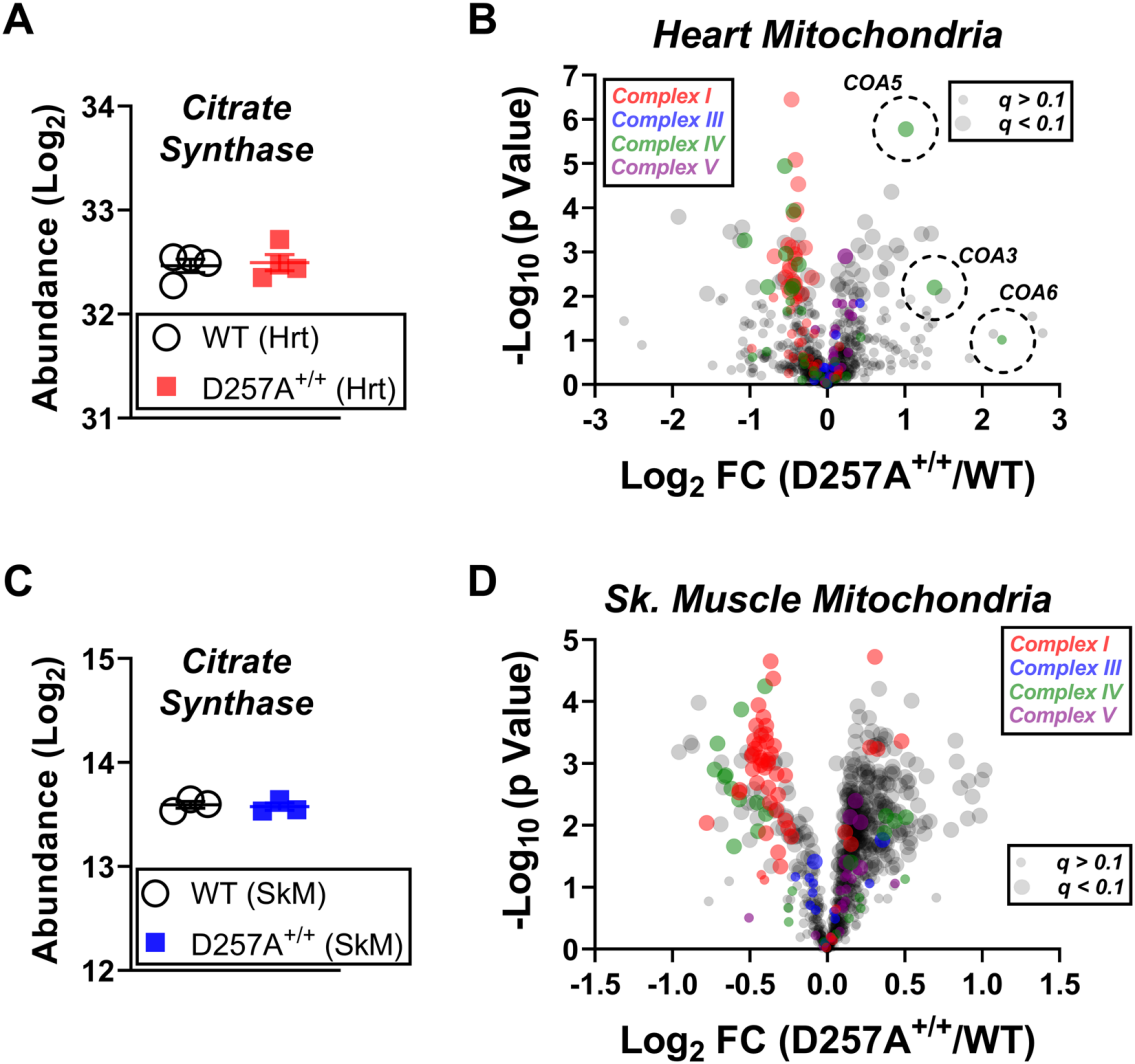
Selective loss of respiratory complexes I and IV in D257A^+/+^ heart and skeletal muscle mitochondria. Data were searched using the mouse Mito Carta 2.0 database. (**A**) Protein abundance of citrate synthase in heart mitochondria from WT and D257A^+/+^. (**B**) Volcano plot depicting changes in the heart mitochondrial proteome between genotypes. Protein subunits of each respiratory complex (CI, CIII, CIV, CV) are indicated by color. Significance is indicated by the size of each circle, with ‘significance’ (adjusted p value <0.1) being represented by the larger circles. Dashed circles indicate the CIV assembly factors COA3, COA5, and COA6. (**C**) Protein abundance of citrate synthase in skeletal muscle mitochondria from WT and D257A^+/+^. (**D**) Volcano plot depicting changes in the skeletal muscle mitochondrial proteome between genotypes. Protein subunits of each respiratory complex (CI, CIII, CIV, CV) are indicated by color. Significance is indicated by the size of each circle, with ‘significance’ (adjusted p value <0.1) being represented by the larger circles.

When performing subcellular fractionation, previous reports have demonstrated that this technique does not produce 100% pure mitochondrial preps^13^. To control for group differences in mitochondrial versus non-mitochondrial protein abundance, nLC-MS/MS data were re-searched using the MitoCarta 2.0 database^14^, to effectively normalize protein expression to apparent mitochondrial content. Importantly, citrate synthase abundance was identical across genotypes using this approach, confirming equal total amounts of mitochondrial protein across samples (Fig. 1A). Of the 790 identified and quantified mitochondrial proteins, 77 were differentially expressed in D257A^+/+^ cardiac mitochondria (Supplementary Table 2). While 48 proteins were downregulated in D257A^+/+^ mitochondria, over half of these proteins (29 total) corresponded to subunits of CI or CIV of the respiratory system (Fig. 1B). Despite mtDNA encoding protein subunits for all four of the main transmembrane respiratory complexes, not a single protein subunit of complex III (CIII) or complex V (CV) was decreased in expression in D257A^+/+^ heart mitochondria. Interestingly, the CIV assembly factors COA5 and COA3 were both upregulated in D257A^+/+^ cardiac mitochondria (Fig. 1B).

**Figure 2:**
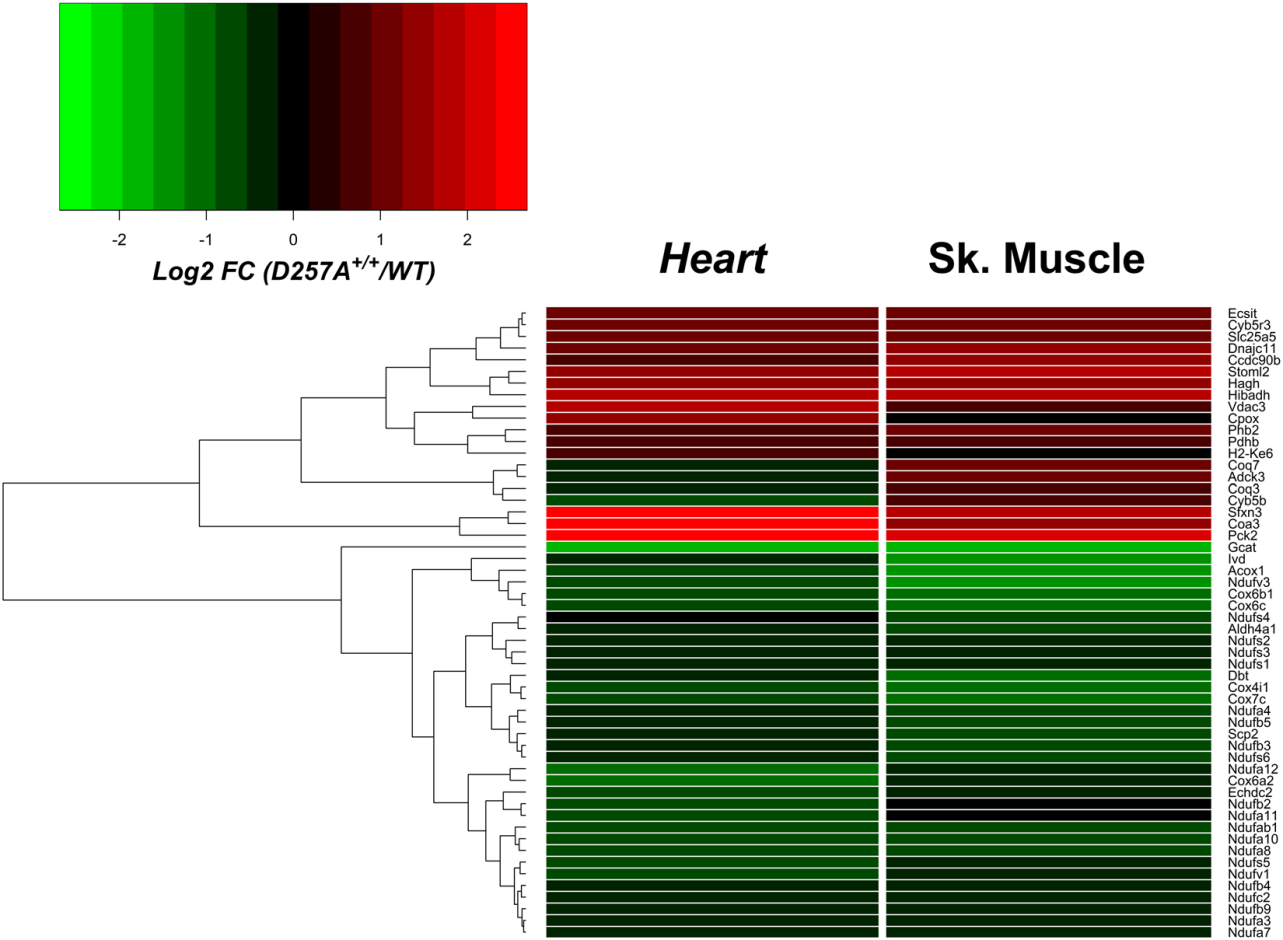
Common differentially expressed proteins across heart and skeletal muscle mitochondria of D257A^+/+^ mice. Heat map displaying the common differentially expressed proteins (adjusted p value <0.1) across the mitochondrial proteome of D257A^+/+^ heart and skeletal muscle. Data are displayed as Log_2_ fold change (D257A^+/+^/WT).

To determine if the effects observed in cardiac mitochondria were also present in other tissues, we conducted an additional proteomics screen using TMT-labeled peptides from skeletal muscle mitochondria (Supplemental Table 3). Citrate synthase abundance was once again identical across genotypes (Fig. 1C). Compared to CIII and CV, protein subunits of both CI and CIV were highly enriched across all downregulated targets (adjusted p value <0.1) in D257A^+/+^ skeletal muscle mitochondria (Fig. 1D). Direct comparison across tissues revealed 54 shared differentially expressed protein targets in D257A^+/+^ mitochondria, bookended by glycine acetyltransferase (GCAT, downregulated) and phosphoenolpyruvate carboxykinase (PCK2, upregulated) (Fig. 2, Supplementary Table 3). Taken together, these data corroborate the previously published bioenergetic phenotype of D257A^+/+^ mitochondria and indicate that the primary impact of dysfunctional mitochondrial polymerase encompasses a specific reduction in CI and CIV^10^.

### D257A^+/+^ induced alterations in mtDNA encoded protein abundances is heterogeneous across the 13 respiratory complex proteins

**Figure 3:**
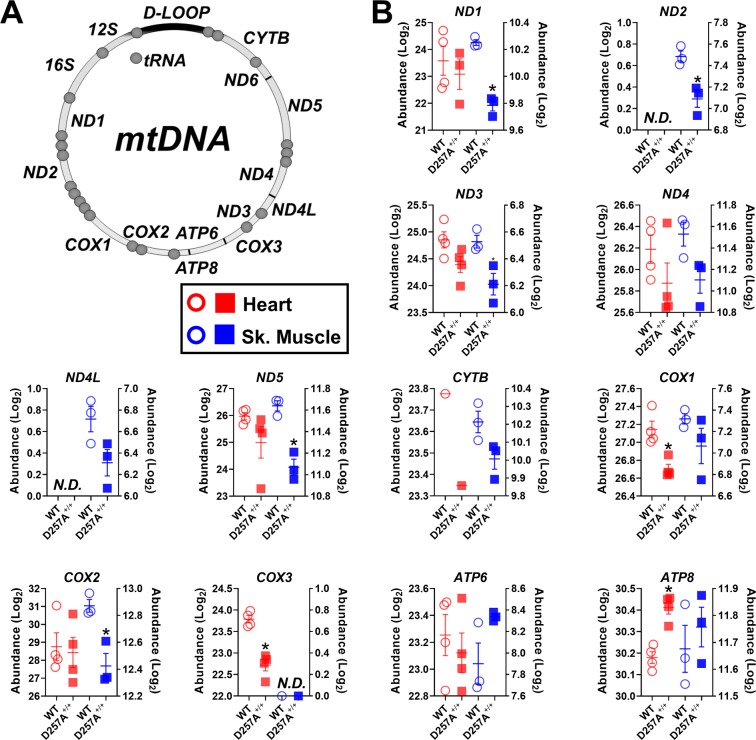
Effect of compromised mitochondrial polymerase activity on the protein abundance of mtDNA-encoded genes. **(A)** Cartoon depicting mammalian mtDNA. (**B**) Protein abundance (Log_2_) of 12 of the 13 mtDNA genes broken down by tissue (ND1, ND2, ND3, ND4, ND4L, ND5, CYTB, COX1, COX2, COX3, ATP6, ATP8). Heart protein abundance corresponds to the left y-axis, while skeletal muscle protein abundance is reflected by the right y-axis. *Adjusted p value <0.1. Data are Mean ± SEM, N = 4/group (heart), N = 3/group (skeletal muscle).

The mtDNA encodes 13 protein-coding genes, corresponding to 7 subunits of CI (ND1, ND2, ND3, ND4, ND4L, ND5, ND6), 1 subunit of CIII (CYTB), 3 subunits of CIV (COX1, COX2, COX3), and 2 subunits of CV (ATP6, ATP8) (Fig. 3A). Although only ND1, ND2, ND5, COX1, COX2, and COX3 were significantly downregulated in D257A^+/+^ mitochondria across tissues, the other CI (ND3, ND4, ND4L) subunits tended to be lower in expression, as was CytB (Fig. 3B). Interestingly, expression of both CV subunits (ATP6 and ATP8) was either unchanged or increased in D257A^+/+^ mitochondria (Fig. 3B). Together, these data suggest that the mutation-associated loss of mtDNA-encoded protein abundance in the setting of dysfunctional proofreading activity is preferentially concentrated to specific genetic loci, particularly those corresponding to ND1, ND2, ND5, COX1, COX2, and COX3.

### Protein phosphatase 1 K (PPM1K) expression predicts respiratory limitations in aged cardiac mitochondria

**Figure 4:**
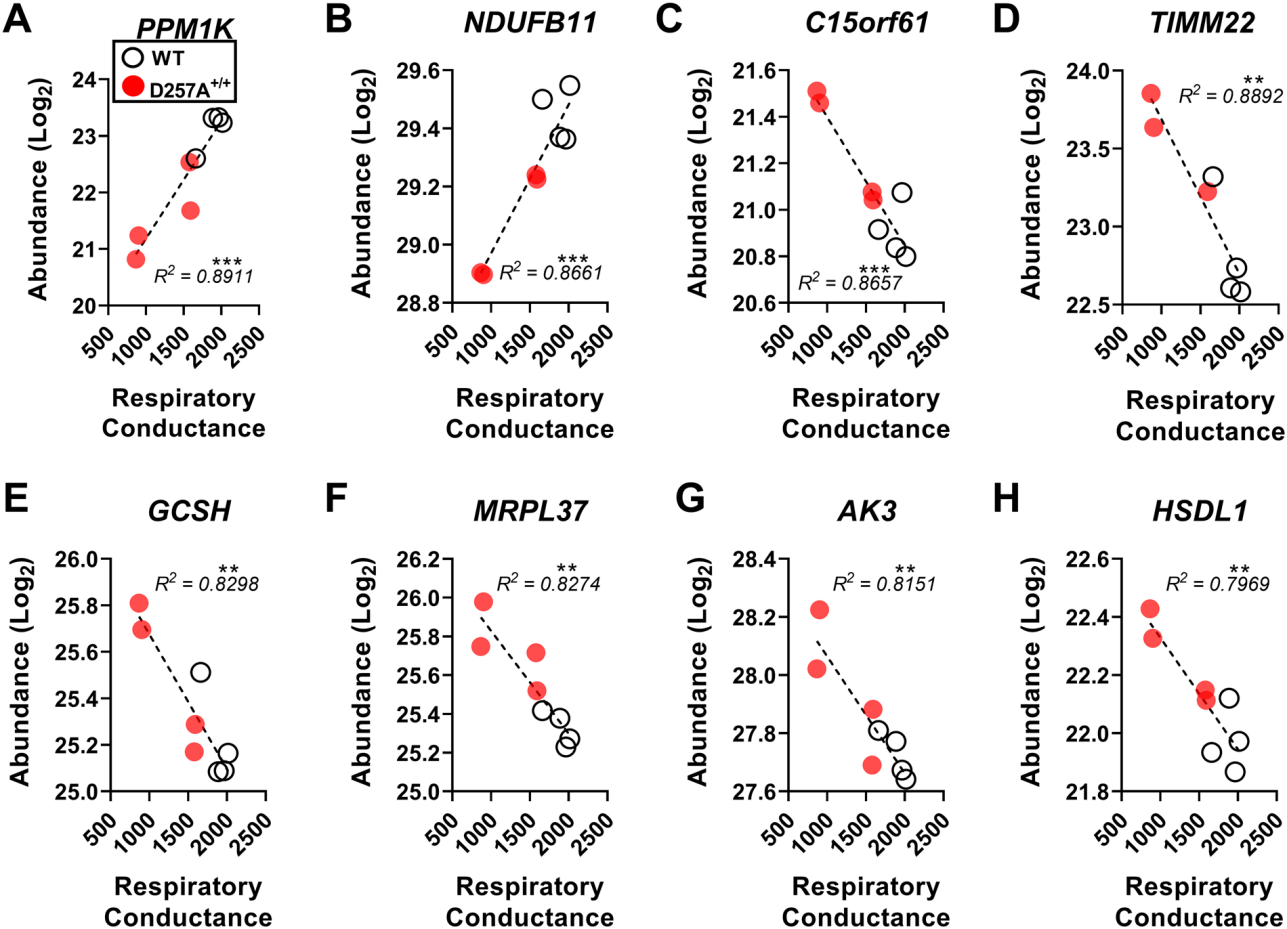
Relationship between respiratory conductance and protein abundance in WT and D257A^+/+^ heart mitochondria. Correlation of respiratory conductance with the abundance of (**A**) PPM1K, (**B**) NDUFB11, (**C**) C15orf61, (**D**) TIMM22, (**E**) GCSH, (**F**) MRPL37, (**G**) AK3, (**H**) HSDL1. **P < 0.01, ***P < 0.001, N = 8 (D257A^+/+^ samples indicated in ‘Red’).

We recently applied a comprehensive mitochondrial phenotyping platform to investigate the bioenergetic consequences of a proofreading-deficient mitochondrial polymerase in D257A^+/+^ cardiac mitochondria^10^. Results revealed substrate-independent impairments in respiratory conductance in D257A^+/+^ mitochondria, likely secondary to respiratory complex downregulation. To investigate potential adaptations across the mitochondrial proteome in response to respiratory insufficiency, we performed a correlation analysis on all identified mitochondrial proteins against the previously reported individual measurements of global respiratory conductance. Of the 791 quantified mitochondrial proteins, 35 were significantly (P < 0.01) correlated with respiratory conductance (Fig. 4A–H; Supplemental Table 4). Remarkably, PPM1K was found to be the greatest predictor of respiratory insufficiency, particularly in D257A^+/+^ mitochondria (Fig. 4A). Additional protein targets highly correlated (R^2^ ≥ 0.8) with respiratory conductance included NDUFB11, C15orf61, TIMM22, GCSH, MRPL37, AK3, and HSDL1 (Fig. 4B–H). Together, these data highlight the power of comprehensive bioenergetic phenotyping alongside discovery-based proteomics and identify PPM1K as a potential indicator of respiratory limitations in heart mitochondria.

## Discussion

This study characterized the mitochondrial-enriched proteomes of the heart and skeletal muscle of the mtDNA mutator mouse. Mitochondrial enrichment was successful as most proteins that were identified and quantified were known to be localized to the mitochondrial compartment. Further analysis was restricted to the mitochondrial proteome (based on the Mito Carta 2.0 database) as the present study was specifically designed to identify protein predictors (i.e., biomarkers) of bioenergetic insufficiency across healthy (WT) and compromised mitochondria (D257A^+/+^). By correlating proteomic and respiratory conductance data from heart mitochondria isolated from the same cohort of mice, novel relationships between heart protein expression and respiratory insufficiency were identified that may represent biomarkers for bioenergetic stress.

Among the proteins most significantly downregulated in D257A^+/+^ heart and skeletal muscle mitochondria were subunits corresponding to CI and CIV, including both nuclear and mitochondrial genome-encoded proteins. Specific reductions in CI and CIV subunits are consistent with previous proteomic assessments of the skeletal muscle and brain tissue of D257A^+/+^ mice^10–12^. Given that CI and CIV have the greatest proportional gene representation in the mtDNA of any of the respiratory complexes, it is possible that they may also have the greatest susceptibility to mtDNA mutation caused by loss of proofreading activity in the mitochondrial polymerase. With respect to only those genes encoded within the mtDNA, as might be expected, the majority of proteins were trending toward decreased abundance in both heart and skeletal muscle of D257A^+/+^. It should be noted that the limited sample size of the current study may have masked potential decreases in protein abundance across the respiratory complexes, as the present study was not powered to detect smaller, more nuanced, differences in protein expression. Interestingly, the two mitochondrial-encoded protein subunits of complex V (i.e., ATP synthase) were unchanged or significantly upregulated in both tissues compared to WT. Reduced CIV subunit expression paired with preserved or increased CV expression has previously been found in the liver and heart tissue of D257A^+/+^ mice^5^. This may be due to the fact that the genes encoding these proteins are shorter in length than some of the other mtDNA protein-encoding genes, particularly those of CI and CIV. In support of this notion, Gasparre *et al*.^15^ reported that the mutational burden within the mtDNA was not equivalent across the 13 genes. In fact, the mitochondrial-encoded CI genes were among the most likely to be pathogenically mutated (i.e., a mutation that impinges on protein function). In contrast, the CV subunits were among the least likely to display pathogenic mutations. Although their analysis was completed using mtDNA mutations present in oncocytic tumors, the present data would suggest that to some extent the mtDNA-encoded CV protein subunits are also robust to mutation in D257A^+/+^ mitochondria.

Correlating heart proteomic data to previous measurements of respiratory conductance in WT and D257A^+/+^ heart mitochondria yielded several strong potential predictors of respiratory insufficiency. Of note, abundance of the CI accessory subunit NDUFB11 was the strongest respiratory complex protein predictor of biochemical conductance, reflecting the general loss of CI subunits that was seen in D257A^+/+^ mitochondria. Interestingly, the protein with the most significant correlation to respiratory conductance was PPM1K, a serine/threonine phosphatase that has been implicated in both branched-chain amino acid metabolism and the mitochondria permeability transition^16,17^. While speculative, the strong correlation between PPM1K and respiratory conductance suggests that, in addition to changes to protein expression, there may be adaptations to the post-translational modification landscape (e.g., phosphoproteome) in response to chronic bioenergetic insufficiency. In addition to PPM1K, C15orf61, a protein with no known function, had a significant negative correlation with respiratory conductance. Rounding out the top eight most highly correlated proteins were TIMM22 (mitochondrial protein import), GCSH (glycine metabolism), MRPL37 (mitochondrial ribosomal protein), AK3 (nucleoside metabolism), and HSDL1 (inactive hydroxysteroid dehydrogenase-like protein). The implications of these correlations remain unclear but warrant further investigation in other tissues and/or states of respiratory insufficiency to confirm their utility as adaptations to respiratory limitations or bona fide biomarkers of mitochondrial stress.

The results of this study have confirmed that the respiratory insufficiency observed in the D257A^+/+^ mouse can be linked to reduced expression of both the mitochondrial and nuclear encoded subunits of respiratory complexes I and IV. The bioenergetic limitations of D257A^+/+^ mitochondria are evident despite the preserved expression of CV, and likely contribute to the accelerated aging phenotype and cardiac pathology previously reported in the mutator mouse line^2,6^. Taken together, the present findings highlight the diagnostic power of compressive bioenergetic phenotyping combined with discovery proteomics to identity unique signatures of bioenergetic stress across the mitochondrial network.

## Methods

All animal studies were performed in accordance with the relevant guidelines and regulations and approved by the East Carolina University Institutional Animal Care and Use Committee. Homozygous PolG mutants (D257A^+/+^) and wild-type (D257A^−/−^, forward known as WT) littermates, on a C57BL/6J background, were aged to 12-13 months prior to experimentation. These mice display an accelerated aging phenotype, due to an accumulation of mtDNA mutations^2–5^. All mice were housed in a temperature (22 °C) and light controlled (12 h light/12 h dark) room and given free access to food and water. At the time of tissue harvest, mice were anesthetized with isofluorane, and hearts (complete left and right ventricles) and skeletal muscle (tibialis anterior muscles from both legs) were removed and subjected to mitochondrial isolation immediately (heart) or flash frozen and later subjected to mitochondrial isolation (skeletal muscle).

### Chemical & reagents

Unless otherwise stated, all chemicals were purchased from Sigma-Aldrich.

### Mitochondrial isolation

Mitochondria were isolated as described previously^10^. Differential centrifugation was employed to prepare isolated mitochondria from heart and skeletal muscle. The following buffers were utilized: Buffer A - MOPS (50 mM; pH = 7.1), KCl (100 mM), EGTA (1 mM), MgSO4 (5 mM); Buffer B - Buffer A, supplemented with bovine serum albumin (BSA; 2 g/L). Hearts were excised and immediately placed in ice-cold Buffer B. Flash frozen skeletal muscle samples were thawed in ice-cold Buffer B. All tissues were minced and then homogenized via a Teflon pestle and borosilicate glass vessel. Tissue homogenates were centrifuged at 600 × g for 10 minutes at 4 °C. Supernatant was filtered through thin layers of gauze and subjected to an additional centrifugation at 10,000 × g for 10 minutes at 4 °C. Mitochondrial pellets were washed in 1.4 ml of Buffer A, transferred to microcentrifuge tubes and again centrifuged at 10,000 × g for 10 minutes at 4 °C. Buffer A was aspirated from each tube and final mitochondrial pellets were suspended in 100–200 μL of Buffer A. Protein content was determined via the Pierce BCA protein assay. Functional assays in heart mitochondria were carried out in Buffer C - Potassium-MES (105 mM; pH = 7.2), KCl (30 mM), KH_2_PO_4_ (10 mM), MgCl_2_ (5 mM), EGTA (1 mM), BSA (2.5 g/L). Mitochondrial pellets not used for functional experiments were flash frozen and later subjected to proteomics analysis.

### Mitochondrial respiratory control

High-resolution O_2_ consumption measurements were conducted using the Oroboros Oxygraph-2K (Oroboros Instruments). All experiments were carried out at 37 °C in a 2 mL reaction volume. Steady-state oxygen consumption rates (*J*O_2_) were determined within individual experiments using a modified version of the creatine kinase energetic clamp technique^18,19^. In this assay, the free energy of ATP hydrolysis (ΔG′_ATP,_ depicted throughout the manuscript as ΔG_ATP_) can be calculated based on known amounts of creatine (Cr), phosphocreatine (PCr) and ATP in combination with excess amounts of creatine kinase (CK) and the equilibrium constant for the CK reaction (i.e., *K*_CK_). Calculation of ΔG′_ATP_ was performed according to the following formula using the previously published online resource (https://dmpio.github.io/bioenergetic-calculators/ck_clamp/)^20^:

$$\Delta {G}{{\prime} }_{{\rm{ATP}}}=\Delta {G}{{\prime} }_{{\rm{ATP}}}^{^\circ }+{RT}\,\mathrm{ln}\,\frac{[{\rm{Cr}}][{P}_{{i}}]}{[{\rm{PCr}}][{K}{{\prime} }_{{\rm{CK}}}]}$$

where ΔG′°_ATP_ is the standard apparent transformed Gibbs energy (under a specified pH, ionic strength, free magnesium and pressure), R is the gas constant (8.3145 J/kmol) and T is temperature in kelvin (310.15). Given that experiments were performed via sequential additions of PCr, both the ΔG′°_ATP_ and *K*’_CK_ were determined at each titration step based on the changes in buffer ionic strength and free magnesium, as previously described^21,22^. Buffer for all assays was Buffer C, supplemented with ATP (5 mM), Cr (5 mM), PCr (1 mM), and CK (20 U/mL). To begin, isolated mitochondria (0.05 mg/mL) were added to assay buffer, followed by the addition of respiratory substrates. The following substrate conditions were tested: pyruvate/malate (5/2.5 mM), glutamate/malate (10/2.5 mM) palmitoyl-carnitine/malate (0.02/2.5 mM), succinate/rotenone (10/0.005 mM). Following substrate additions, sequential additions of PCr to 6, 9,15 and 21 mM were performed to gradually slow *J*O_2_ back toward baseline. Plotting the calculated ΔG_ATP_ against the corresponding *J*O_2_ reveals a linear force-flow relationship, the slope of which represents the conductance/sensitivity of the entire respiratory system under specified substrate constraints as previously described^20^. For each sample, respiratory conductance values from all substrates were pooled and subsequently correlated with protein expression across the mitochondrial proteome.

### Mitochondrial lysis, protein digestion, and peptide labeling for label-free (heart) or TMT (skeletal muscle) quantitative proteomics

Heart mitochondrial pellets, as well as skeletal muscle mitochondrial pellets (approximately 250 µg of protein) from D257A^+/+^ and WT mice (n = 4/group – heart; n = 3/group – skeletal muscle) were lysed in ice-cold 8 M Urea Lysis Buffer (8 M urea in 40 mM Tris, pH 8.0, 30 mM NaCl, 1 mM CaCl_2_, 1x cOmplete ULTRA mini EDTA-free protease inhibitor tablet), as described previsouly^23^. The Samples were frozen on dry ice and thawed for three freeze-thaw cycles and further disrupted by sonication with a probe sonicator in three 5 second bursts (Q Sonica #CL-188; amplitude of 30). Samples were centrifuged at 10,000 × g for 10 min at 4 °C to pellet insoluble material. Protein concentration was determined by BCA, and equal amounts of protein (200 μg, adjusted to 2.5 mg/mL with Urea Lysis Buffer) were reduced with 5 mM DTT at 37 °C for 30 min, cooled to room temperature, and then alkylated with 15 mM iodoacetamide for 30 min in the dark. Unreacted iodoacetamide was quenched by the addition of DTT up to 15 mM. Initial digestion was performed with Lys C (ThermoFisher Cat# 90307; 1:100 w-w; 2 ug enzyme per 200 ug protein) for 4 hours at 37 °C. Following dilution to 1.5 M urea with 40 mM Tris (pH 8.0), 30 mM NaCl, 1 mM CaCl_2_, the samples were digested overnight with trypsin (Promega; Cat# V5113; 50:1 w/w, protein:enzyme) at 37 °C. Samples were acidified to 0.5% TFA and centrifuged at 4000 × g for 10 min at 4 °C to pellet insoluble material. Supernatant containing soluble peptides was desalted on a 50 mg tC18 SEP-PAK solid phase extraction column (Waters; Cat# WAT054955) and eluted (500 μL 25% acetonitrile/0.1% TFA and 2 × 500 μL 50% acetonitrile/0.1% TFA). The 1.5 mL eluate was frozen and lyophilized.

### TMT labeling

TMT labeling was performed as previously described^23^. The six samples from skeletal muscle isolated mitochondria were re-suspended in 100 μL of 200 mM triethylammonium bicarbonate (TEAB), mixed with a unique 6-plex Tandem Mass Tag (TMT) reagent (0.8 mg re-suspended in 50 μL100% acetonitrile), and shaken for 4 hours at room temperature (ThermoFisher Scientific; Cat# 90064). Following quenching with 0.8 μL 50% hydroxylamine, all six samples from each tissue were combined, frozen, and lyophilized. Samples were re-suspended in ~1 mL of 0.5% TFA and again subjected to solid phase extraction, but with a 100 mg tC18 SEP-PAK SPE column (Waters; Cat# WAT023590). The multiplexed peptide sample was subjected to high pH reversed phase fractionation according to the manufacturer’s instructions (ThermoFisher Cat# 84868). In this protocol, peptides (100 µg) are loaded onto a pH-resistant resin and then desalted with water washing combined with low speed centrifugation. A step-gradient of increasing acetonitrile concentration in a high-pH elution solution is then applied to columns to elute bound peptides into 8 fractions. Following elution, fractions were frozen and lyophilized.

### nLC-MS/MS for label-free proteomics

As described previously^23^, with some modification, all samples were suspended in 0.1% formic acid at a concentration of 0.25 µg/µL, following peptide quantification (ThermoFisher Cat# 23275). Samples were subjected to *nano*LC-MS/MS analysis using an UltiMate 3000 RSLCnano system (ThermoFisher) coupled to a *Q Exactive Plus* Hybrid Quadrupole-Orbitrap mass spectrometer (ThermoFisher) *via* a nanoelectrospray ionization source. For each injection of 4 µL (1 µg), the sample was first trapped on an Acclaim PepMap^TM^ 100 20 mm × 0.075 mm trapping column (ThermoFisher Cat# 164535; 5 μl/min at 98/2 v/v water/acetonitrile with 0.1% formic acid), after which the analytical separation was performed over a 95-minute gradient (flow rate of 250 nanoliters/minute) of 4 to 30% acetonitrile using a 2 µm EASY-Spray PepMap^TM^ RSLC C18 75 µm × 250 mm column (ThermoFisher Cat# ES802A) with a column temperature of 35 °C. MS1 was performed at 70,000 resolution, with an AGC target of 3 × 10^6^ ions and a maximum injection time (IT) of 100 ms. MS2 spectra were collected by data-dependent acquisition (DDA) of the top 15 most abundant precursor ions with a charge greater than 1 per MS1 scan, with dynamic exclusion enabled for 30 seconds. Precursor ions were filtered with a 1.5 m/*z* isolation window and fragmented with a normalized collision energy of 27. MS2 scans were performed at 17,500 resolution, AGC target of 1 × 10^5^ ions, and maximum IT of 50 ms.

### nLC-MS/MS for TMT proteomics

As described previously^23^, with some modification, peptide fractions were suspended in 0.1% formic acid at a concentration of 0.25 µg/µL, following peptide quantification (ThermoFisher Cat# 23275). All samples were subjected to *nano*LC-MS/MS analysis using an UltiMate 3000 RSLCnano system (ThermoFisher) coupled to a *Q Exactive Plus* Hybrid Quadrupole-Orbitrap mass spectrometer (ThermoFisher) *via* a nanoelectrospray ionization source. For each injection of 4 µL (1 µg), the sample was first trapped on an Acclaim PepMap^TM^ 100 20 mm × 0.075 mm trapping column (ThermoFisher Cat# 164535; 5 μl/min at 98/2 v/v water/acetonitrile with 0.1% formic acid), after which the analytical separation was performed over a 90-minute gradient (flow rate of 300 nanoliters/minute) of 3 to 30% acetonitrile using a 2 µm EASY-Spray PepMap^TM^ RSLC C18 75 µm × 250 mm column (ThermoFisher Cat# ES802A) with a column temperature of 55 °C. MS1 was performed at 70,000 resolution, with an AGC target of 1 × 10^6^ ions and a maximum IT of 60 ms. MS2 spectra were collected by data-dependent acquisition (DDA) of the top 20 most abundant precursor ions with a charge greater than 1 per MS1 scan, with dynamic exclusion enabled for 30 seconds. Precursor ions were filtered with a 1.0 m/*z* isolation window and fragmented with a normalized collision energy of 30. MS2 scans were performed at 17,500 resolution, AGC target of 1 × 10^5^ ions, and a maximum IT of 60 ms.

### Data analysis for label-free proteomics

As described previously^23^, with some modification, Proteome Discoverer 2.2 (PDv2.2) was used for raw data analysis, with default search parameters including oxidation (15.995 Da on M) as a variable modification and carbamidomethyl (57.021 Da on C) as a fixed modification, and 2 missed cleavages (full trypsin specificity). Data were searched against the Uniprot mouse proteome database, as well as the mouse Mito Carta 2.0 database^14^. PSMs were filtered to a 1% FDR. PSMs were grouped to unique peptides while maintaining a 1% FDR at the peptide level. Peptides were grouped to proteins using the rules of strict parsimony and proteins were filtered to 1% FDR using the Protein FDR Validator node of PD2.2. Peptide quantification was done using the MS1 precursor intensity. Imputation was performed *via* low abundance resampling.

### Data analysis for TMT proteomics

As described previously^23^, with some modification, Proteome Discoverer 2.2 (PDv2.2) was used for raw data analysis, with default search parameters including oxidation (15.995 Da on M) as a variable modification and carbamidomethyl (57.021 Da on C) and TMT6plex (229.163 Da on peptide N-term and K) as fixed modifications, and 2 missed cleavages (full trypsin specificity). Data were searched against the Uniprot mouse proteome database, as well as the mouse Mito Carta 2.0 database^14^. PSMs were filtered to a 1% FDR. PSMs were grouped to unique peptides while maintaining a 1% FDR at the peptide level. Peptides were grouped to proteins using the rules of strict parsimony and proteins were filtered to 1% FDR using the Protein FDR Validator node of PD2.2. MS2 reporter ion intensities for all PSMs having co-isolation interference below 0.5 (50% of the ion current in the isolation window) and an average S/N > 10 for reporter ions were summed together at the peptide and protein level. Imputation was performed *via* low abundance resampling.

### Statistical analysis for label-free and TMT proteomic

Protein and peptide groups tabs in the PDv2.2 results were exported as tab delimited.txt. files, and analyzed based on a previously described workflow^23,24^, with some modification. First, peptide group M1 precursor (label-free) or M2 reporter (TMT) intensities for each peptide group were summed together for each sample or TMT channel, each sample/channel’s sum was divided by the average of all samples/channels’ sums, resulting in sample/channel-specific loading control normalization factors to correct for any deviation from equal protein input in the label-free or six-plex experiments. Precursor or reporter intensities for proteins were divided by the loading control normalization factors for each respective sample or TMT channel. All loading control-normalized precursor or reporter intensities were converted to log_2_ space. For D257A^+/+^ and WT comparisons within each tissue, condition average, standard deviation, p-value (p, two-tailed student’s t-test, assuming equal variance), and adjusted p-value (P_*adjusted*_, Benjamini Hochberg FDR correction) were calculated^25,26^. For protein-level quantification, only Master Proteins—or the most statistically significant protein representing a group of parsimonious proteins containing common peptides identified at 1% FDR—were used for quantitative comparison.

### Proteomics data availability and software

All raw data for proteomics experiments is available online using accession number “PXD017000” for Proteome Xchange^27^ and accession number “JPST000729” for jPOST Repository^28^. Data are presented as mean ± SEM. Figures were generated using GraphPad Prism (Version 8.0). Heat maps were generated using RStudio. Statistical details of each experiment are located in the figure legends. The number of mice per experiment is represented by “N”.

## Supplementary information

Supplementary Information.

Supplementary Table 1.

Supplementary Table 2.

Supplementary Table 3.

Supplementary Table 4.
