# Supplementary material for: Subcellular proteomics combined with bioenergetic phenotyping reveals protein biomarkers of respiratory insufficiency in the setting of proofreading-deficient mitochondrial polymerase
Source: Sci Rep. 2020 Feb 27;10:3603. doi: 10.1038/s41598-020-60536-y (PMC7046634; doi:10.1038/s41598-020-60536-y)
Supplement: Supplementary file 2 — Supplementary Table 1. [file 41598_2020_60536_MOESM2_ESM.pdf]

**Subcellular proteomics combined with bioenergetic phenotyping reveals protein biomarkers of respiratory insufficiency in the setting of proofreading-deficient mitochondrial polymerase.**

Kelsey L. McLaughlin[a,d], Kimberly A. Kew[b], Joseph M. McClung[a,c,d] and Kelsey H. Fisher-Wellman[a,d]*

[a]Department of Physiology, [b]Department of Biochemistry, [c]Department of Cardiovascular Sciences, Brody School of Medicine, East Carolina University, Greenville, NC 27834 USA
[d]East Carolina Diabetes and Obesity Institute, East Carolina University, Greenville, NC 27834 USA

* To whom correspondence should be addressed:
Kelsey H. Fisher-Wellman fisherwellmank17@ecu.edu
East Carolina Diabetes and Obesity Institute, 115 Heart Drive, Greenville, NC 27834 USA
Telephone: 252-744-2585
Fax: 252-744-0462

**SUPPLEMENTAL FIGURES**

**Supplemental Figure 1.**

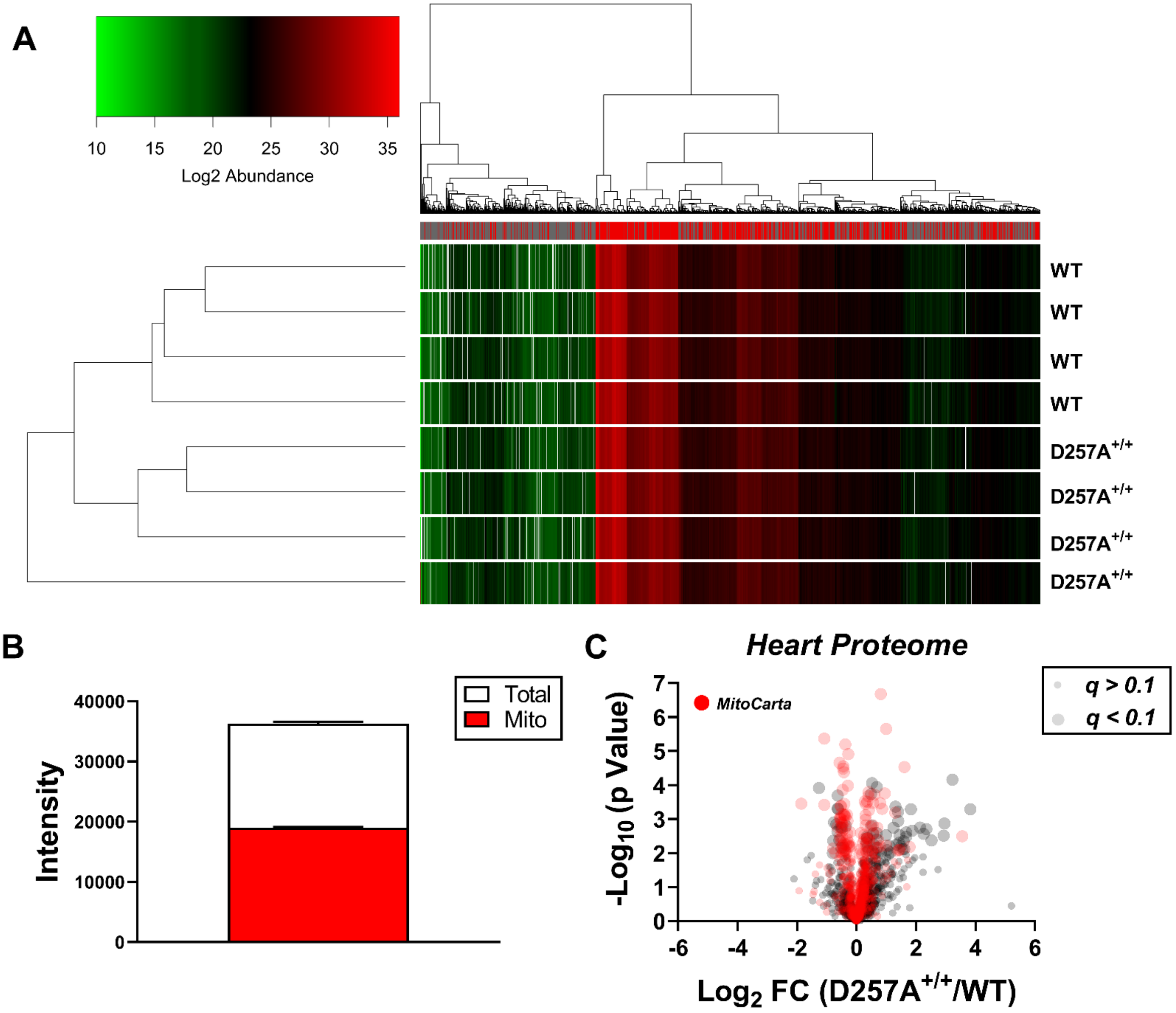

**Supplemental Figure 1. Characterization of the mitochondrial proteome of WT and D257A$^{+/+}$ hearts using differential centrifugation.** (**A**) Label-free nLC-MS/MS was performed on cardiac mitochondrial lysates from WT and D257A$^{+/+}$ mitochondrial pellets. Heat map displaying $Log_2$ protein intensity of all quantified master proteins. Mitochondrial proteins are indicated by the horizontal bar at the top of the heat map in 'Red', non-mitochondrial proteins are indicated as 'Grey'. Mitochondrial assignment was done using the Mito Carta 2.0 database. (**B**) Summed protein intensity of all proteins identified, with mitochondrial proteins indicated in 'Red'. (C) Volcano plot depicting changes in the heart proteome between genotypes. Mitochondrial proteins are indicated in 'Red'. Significance is indicated by the size of each circle, with 'significance' (adjusted p value < 0.1) being represented by the larger circles. N=4/group.

**SUPPLEMENTAL TABLES**

**Supplemental Table 1.** Full proteomics dataset from isolated heart mitochondria. Data was searched using a fasta database corresponding to the entire mouse proteome. (**A**) Exported data from Proteome Discoverer 2.0 ('Peptide Groups'). (**B**) Exported data from Proteome Discoverer 2.0 ('Proteins'). In exported data files (**A-B**), genotype is indicated by 'WT' for wild-type littermates or 'PolG' for D257A$^{+/+}$. (**C**) Complete annotated dataset. Columns A-AB correspond to the following: Accession (Uniprot); Description; M1 Precursor Ion Abundance; M1 Precursor Ion Abundance (normalized to total peptide amount and $Log_2$ transformed); Group Means; $Log_2$ FC (D257A/WT), p value from two-sided t-tests; $-log_{10}$ p value; adjusted p value (< 0.1 indicated by bold text); Mito Carta 2.0 protein (1 = yes); Description.

**Supplemental Table 2.** Full proteomics dataset from isolated heart mitochondria. Data was searched using a fasta database corresponding to Mito Carta 2.0. (**A**) Exported data from Proteome Discoverer 2.0 ('Peptide Groups'). (**B**) Exported data from Proteome Discoverer 2.0 ('Proteins'). In exported data files (**A-B**), genotype is indicated by 'WT' for wild-type littermates or 'PolG' for D257A$^{+/+}$. (**C**) Complete annotated dataset. Columns A-Z correspond to the following: Accession (Uniprot); Description; M1 Precursor Ion Abundances; M1 Precursor Ion Abundances (normalized to total peptide amount and $Log_2$ transformed); Group Means; $Log_2$ FC (D257A/WT), p value from two-sided t-tests; $-log_{10}$ p value; adjusted p value (< 0.1 indicated by bold text); Gene Name.

**Supplemental Table 3.** Full proteomics dataset from isolated skeletal muscle mitochondria. Data was searched using a fasta database corresponding to Mito Carta 2.0. (**A**) Exported data from Proteome Discoverer 2.0 ('Peptide Groups'). (**B**) Exported data from Proteome Discoverer 2.0 ('Proteins'). In exported data files (**A-B**), genotype is indicated by 'WT' for wild-type littermates or 'PolG' for D257A$^{+/+}$. (**C**) Complete annotated dataset. Columns A-V correspond to the following: Accession (Uniprot); Description; M2 Reporter Ion Abundances; M2 Reporter Ion Abundances (normalized to total peptide amount and $Log_2$ transformed); Groups Means; $Log_2$ FC (D257A/WT), p value from two-sided t-tests; $-log_{10}$ p value; adjusted p value (< 0.1 indicated by bold text); Gene Name. (**D**) Common differentially expressed proteins in heart and skeletal muscle mitochondria from D257A$^{+/+}$ mice. Columns A-E correspond to the following: Gene Name; Heart $Log_2$ FC (D257A/WT); Heart adjusted p value; skeletal muscle (SkM) $Log_2$ FC (D257A/WT); SkM adjusted p value.

**Supplemental Table 4.** Relationship between respiratory conductance and the mitochondrial proteome in isolated heart mitochondria from WT and D257A$^{+/+}$ mice. (**A**) Sample Key indicating the following information per sample: Experiment Date – date isolated mitochondria were prepared; Mouse ID – internal mouse identifier; Genotype - genotype is indicated by 'WT' for wild-type littermates or 'PolG' for D257A$^{+/+}$; Raw File Name; Sample Name (PD) – sample name assigned in Proteome Discoverer 2.0. (**B**) Correlations between respiratory conductance and protein abundance. Respiratory conductance for each sample is listed in Row 1, Columns B-I. Columns A-L correspond to the following: Gene Name; M1 Precursor Abundances (normalized to total peptide amount and $Log_2$ transformed); r; $R^2$; p value from two-sided t-test.
